# Context Aware Middleware Architectures: Survey and Challenges

**DOI:** 10.3390/s150820570

**Published:** 2015-08-20

**Authors:** Xin Li, Martina Eckert, José-Fernán Martinez, Gregorio Rubio

**Affiliations:** Research Center on Software Technologies and Multimedia Systems for Sustainability (CITSEM—Centro de Investigación en Tecnologías Software y Sistemas Multimedia Para la Sostenibilidad), Campus Sur UPM, Ctra. Valencia, Km 7, 28031 Madrid, Spain; E-Mails: martina.eckert@upm.es (M.E.); jf.martinez@upm.es (J.-F.M.); grubio@diatel.upm.es (G.R.)

**Keywords:** context, context awareness, context aware middleware, ontology, modelling, reasoning

## Abstract

Context aware applications, which can adapt their behaviors to changing environments, are attracting more and more attention. To simplify the complexity of developing applications, context aware middleware, which introduces context awareness into the traditional middleware, is highlighted to provide a homogeneous interface involving generic context management solutions. This paper provides a survey of state-of-the-art context aware middleware architectures proposed during the period from 2009 through 2015. First, a preliminary background, such as the principles of context, context awareness, context modelling, and context reasoning, is provided for a comprehensive understanding of context aware middleware. On this basis, an overview of eleven carefully selected middleware architectures is presented and their main features explained. Then, thorough comparisons and analysis of the presented middleware architectures are performed based on technical parameters including architectural style, context abstraction, context reasoning, scalability, fault tolerance, interoperability, service discovery, storage, security & privacy, context awareness level, and cloud-based big data analytics. The analysis shows that there is actually no context aware middleware architecture that complies with all requirements. Finally, challenges are pointed out as open issues for future work.

## 1. Introduction

Due to the rapid development and penetration of ubiquitous computing and the Internet of Things (IoT) into daily life, a boom of context data which continually represents changes of the environment is generated and can be available for further use [1]. It is believed that the full utilization of this large volume of context data can introduce possibilities for many new applications. Taking one possibility as an example, context aware applications have attracted a lot of attention from academia and industry. Context aware applications are able to adapt their behaviours to the changing environment with a minimum of human intervention, but meanwhile introduce a new challenge for application developers. Therefore, the underlying challenge is to explore an efficient solution to increase the usability of context.

In general, three typical approaches have been of much value to develop context aware applications [2]: (1) Each application interacts, obtains, processes and uses the context of its interest in its own manner; (2) Some libraries/toolkits aiming at acquiring and processing context can be added and reused for building context aware applications; (3) Applications are built on the basis of context aware middleware in which context management is enabled. The third approach outperforms the other two since it can decrease the complexity of developing context aware applications. Hence, context aware middleware is highlighted as an essential requirement for building context aware applications. Traditional middleware, acting as a software layer, is usually defined adhering to the metaphor of a *black box* which hides the heterogeneity of hardware and eases the development of upper applications. To distinguish from the traditional middleware, context aware middleware should provide fundamental context management, such as modelling context, processing context, *etc.* As a result, applying context aware middleware can free developers from the concern of managing the context and allow them to focus on designing desired application functions and business logic.

Many context aware middleware architectures [3,4,5,6] have been proposed to proactively provide adaptive behaviour to the continually changing environment in recent years. Thus it is valuable to review these recent publications and attempt to derive the development trend(s). However, a literature review on context aware middleware architectures reveals two major shortcomings: one is the lack of published surveys on this topic and their missing extension & comprehensiveness since many of them are outdated and do not include newer context aware middleware proposals. The other shortcoming is the big overlap between those published surveys as they repeatedly investigate the same knowledge base (several well-known context aware middlewares). Due to this background, this paper provides an extensive survey on context aware middleware architectures by means of presentation, comparison and evaluation of newly proposed context aware middleware architectures that have not been mentioned in the review published literature. In this way, readers could refresh their knowledge and stay up on the latest context aware middleware developments. Our aim is to present the current status of context aware middleware, point out the challenges behind as well as potential work in order to help researchers and developers to choose a desired middleware for their own use or probably design a brand-new solution inspired by existing proposals.

The remaining part of this paper is organised as follows: Section 2 provides the background of context aware middleware. Herein, the principles of context, context awareness and their role in the middleware are introduced. Familiar readers could skip this part. Section 3 gives an extensive survey of context aware middleware architectures along with analysis and evaluation. Section 4 gives an outlook of open issues and actual challenges. Finally, Conclusions are presented in Section 5.

## 2. Principles of Context Aware Environments

The middleware, as a software layer to abstract the heterogeneity of the lower layer (e.g., hardware) and ease the complexity of developing a higher layer (e.g., applications), is often proposed to be enhanced with the ability of context awareness. Context aware middleware can be adaptive to the environment and provide relevant services according to the changing needs from the external side (e.g., users).

Before digging into the interior composition of context aware middleware, it is necessary to have a fundamental knowledge base regarding context and context awareness. To this end, an introduction to those preliminary concepts is provided in the following subsections.

### 2.1. Context

Context is the key knowledge source for systems to achieve context awareness. The Oxford Dictionary gives a general definition for context as “*the circumstances that form the setting for an event, statement, or idea and in terms of which it can be fully understood.*” Nevertheless, many researchers try to define context in their own way, depending on the necessities and investigated environment, e.g.,

Context is…

- “…*the set of location, identities of nearby people and objects and changes to those objects*.” [7]
- “…*location, identities of the people around the user, the time of day, season, temperature and so forth*.” [8]
- “…*the combination of the user’s location, environment, identity and time*.” [9]
- “…*what is happening at this moment*.” [10]
- “…*the state of the application’s surroundings*.” [11]
- “…*just the aspects of a current situation*.” [12]
- “…*extending to model the activities and tasks that are taking place in a location*.” [13]
- “…*the set of circumstances surrounding it are of relevance to its completion*.” [14]
- “…*any information that can be used to characterize the situation of entities (i.e., whether a person, place, or object) are considered relevant to the interaction between a user and an application, including the user and the application. Context is typically the location, identity, and state of people, groups and computational and physical objects*.” [15]

Taking a holistic view on the aforementioned definitions, it can be found that most of them only focus on a particular application or just enumerate the entities for context. In other words, they lack generality and standardization.

In this paper, we hold the opinion that context is any piece of information that can represent changes of the circumstance (either static or dynamic). Further, it could be useful for understanding the current situation and predicting potential changes.

#### 2.1.1. Context Categorization

A clear and accurate classification of context is helpful to uncover, understand, manipulate and sort out a variety of contexts in an efficient way. Also, it can provide great help for users to identify the type of a given context before using it. As context could be categorized from different perspective, the list in Table 1 summarizes a few well-known forms of context classification given in the current literature [16,17,18,19,20,21,22].

**Table 1:** Context categorizations.

Context Categorization	Observation Aspects and Context Features	Examples
**5W1H (Who, When, Where, What, Why and How)**	Analysis of the environment from different points of view. Intuitive to understand.	**Life assistance of elderly** Peter, 8 o’clock, garden, picking fruit, apples are ripe, with a ladder.
**Physical/Virtual**	Differentiation according to sources: sensing devices (physical); user, context servers, *etc.* (virtual). Simple but ambiguity to identify the same context as it can be physical or virtual depending on different situations.	**Rehabilitation Physical**: heart rate **Virtual**: patient’s medical history from database
**Static/Dynamic**	Observation over time: always equal (static) or adaptive to changes in the environment (dynamic). Intuitive to understand.	**Plant inspection Static**: the place where a tree grows **Dynamic**: the aspect of the tree due to the current season
**Direct/Indirect **	Differentiation through obtainment complexity, indirect context is more complex to acquire and needs computation, inference *etc.* Simple to identify.	**Birthday Direct**: actual date is the birthday **Indirect**: which birthday is it and does this mean something (e.g., 50th Birthday)
**Sensed, Combined, Inferred and Learned**	Refined differentiation of obtainment complexity by sub-categorizations. Ambiguity in identifying the complexity of obtainment, difficult to distinguish clear differences of sub-categorizations	**Navigation Sensed**: proximity to an object **Combined**: speed and direction of motion **Inferred**: check distance (rules) **Learned**: compare with similar situations
**Internal/External**	Differentiation of sources from the user’s point of view. Ambiguity in classifying the same context.	**Life assistance Internal**: desire to get up from the bed **External**: it is the time to get up
**Primary/Secondary**	Obtainment complexity similar to direct/indirect. Simple. Ambiguity in identifying the complexity of obtainment.	**Health monitoring Primary**: check blood pressure **Secondary**: comparison of historical blood pressure data

None of those seven presented types of classification can be called the best or the worst, as each is suitable and reasonable in a certain situation. Hence, depending on certain situation, environment and purpose, a specific classification scheme would be used to handle the context from the desired perspective.

#### 2.1.2. Context Features

It is possible that different kinds of context share several features to some extent. Here, a clear and accurate extraction of context features is beneficial for users to achieve a better understanding of context characteristics so that the shortcomings of context can be minimized or even hidden as much as possible. Van Bunningen *et al.* [23] have identified eight features of context: context is obtained though individual sensors or networks (1); it is sensed by small and constrained devices (2); it originates from distributed sources (3); it is continuously changing (4); it comes from mobile objects (5); it has a temporal (6) and a spatial (7) character and it is imperfect and uncertain (8).

However, from our point of view, some statements are too absolute and inaccurate to be extracted as sharing characteristics for context. Besides, this analysis is not comprehensive enough to represent all aspects of context. So based on this survey, we refine and summarize the following five features for context.

##### Context Sources

Context can be acquired from diverse sources. Generally, obtaining context can be categorized into hardware and virtual sources. From the perspective of hardware, context can be monitored and collected by a variety of sensing devices. The majority of physical context is obtained through sensors or sensor networks. The present trend for hardware development is to become smaller and cheaper from an economic perspective. In the meanwhile, this leads to the restriction of computing capability and storage capacity. Moreover, battery capacity should also be increased to support longer work life. In addition, context can be either provided manually or derived from virtual sources such as context agents, context servers, middleware modules, big data, *etc.*

##### Context Scope

The scope of context types and context data is not limited to a fixed number. On the contrary, it has a wide range and the scope is dynamic, depending on different situations. For instance, a set of information including brightness, temperature, humidity and heart rate can be collected as context in a home healthcare scenario. In addition, the same piece of context information could have different importance in different environments. Due to the fact of enormous context data on behalf of reflecting changes, the raised concerns are computing burden and storage stress.

##### Context Variety

Context can have a lot of alternatives [24]. Different context can reflect similar or even the same changing aspects of a current state so that a big range of flexibility is available for users to choose the most suitable context to use. For example, both address name and latitude/longitude coordinates can record the location of a person. However, the feature results in an extra concern: context redundancy. The increasing context redundancy will pave the way to improve the efficiency of computing capability, extend storage capacity and refine context filtering algorithms, *etc.*

##### Context Temporal Validity

It is known that the majority of context is attributed to the dynamic category. For dynamic context, context data values are only valid for a short period of time until they are replaced by new values. For example, only the real time location is significant for observing a moving person.

##### Context Flexibility

Context is responsive to mobile objects (or persons). Users move around and change from one environment to another which probably is an unfamiliar one. Context data is then collected from the mobile persons and reflects their changes. In this manner, a system is able to accommodate its behavior to each environment. In other words, context is obtained from mobile objects as well as used to serve them in the end.

### 2.2. Context Awareness and Its Categorizations

The term context awareness is often referred to be sentient, reactive, context-sensitive, environment-oriented, situated, responsive and adaptive, it firstly appeared in the Active Badge research project of Olivetti Research Ltd. (Cambridge, UK) in 1992 [25]. From then on, many researchers had interests in generalizing this term and discussing its definition, e.g.,

- “...*the ability of computing devices to detect and sense, interpret and respond to aspects of a user’s local environment and the computing devices themselves*.” [26]
- “...*limited to the human-computer interface* [27], *and the notion of adaptation* [28].”
- “...*provide the maximum flexibility of service based on real time context*.” [29]
- “...*automatically provide information or take actions according to the user’s present context and need*.” [30]
- “...*if an application has the ability to monitor input from sensing devices and choose the suitable context according to user’s need or interests, then it can be labelled as a context-aware application*.” [31]

The authors consider the aforementioned definitions as too one-sided to identify if a system is context aware. As an alternative, the statement found in [22] “*a system is context-aware if it uses context to provide relevant information and/or services to the user, where relevancy depends on the user’s task*” could be viewed as general and accurate enough to suit any context aware application and therefore shared in this paper.

A context aware system is usually designed for a particular purpose or focused on solving certain problems. Therefore, it surely would not be implemented as “all knowing”, but covering necessary aspects. Additionally, differences lie in the degree of awareness which can be expressed as “levels”. For each particular application, those levels have been defined in different ways, so the first aim is to get an overview and then possibly find similarities to achieve a common classification for generally useful levels. Table 2 summarizes different types of classification found in [32,33,34,35] and gives a brief explanation for each level. As can be seen from the Table 2, the focus of each classification is set on very different aspects:

- Observing from the user interaction based viewpoint, the level of context awareness increases as the demand for the invention from users reduces.
- The hardware’s point of view focuses on the acquisition of context data which could be independent (the system itself obtains all necessary data) or based on an external infrastructure of devices. Hereby, intermediate levels could exist, as e.g., context awareness achieved by self-contained hardware could be augmented by using other technologies or infrastructures. The work reported in [32] states a close relation between active context awareness and infrastructure-based context awareness because of some common characteristics. It is believed that the extra infrastructure support is needful for the realization of active context awareness. The prominent commonality is that both try to reduce or even eliminate unnecessary user intervention in order to make the performance as “intelligent” as possible.
- If the measurement by hardware is the only means to realize context awareness, then the corresponding achievement is limited to be in the hard level. Soft context awareness is achieved by means of applying operations, e.g., analysis, inference, and learning on context knowledge base. However, it is difficult to apply and use to examine the level of a real context aware middleware, as most architectures simultaneously depend on both hardware and knowledge repository (context history).
- Based on the different models used to express context, context awareness can be grouped into three levels: location aware, medium and semantic. The investigation of [35] on the history of context aware systems shows that the development went over three generations which happen to represent one level, respectively. The first level is provided merely according to user's location. In the 2nd generation, more kinds of context information are employed to enable systems to know more about the environment. However, due to ambiguity, only a medium level of awareness is reached here. Later, common semantic technologies (e.g., ontology) were adopted to unambiguously represent context structures and their relationships in the 3rd generation. Here, context awareness can take advantage of semantic techniques to ensure formality, flexibility, interoperability and scalability.

**Table 2:** Comparison of context awareness classifications.

Classification Aspect	Context Awareness Levels	Description
**User interaction**	Personalized	Interaction is available for users to set preferences
	Passive	Execution of appropriate options is subject to users’ decisions
	Active	Execution of appropriate options acts autonomously
**Acquisition hardware**	Self-contained	Context awareness is achieved by independent hardware without any external support
	Infrastructure-based	External systems or infrastructures provide additional support to realize context awareness
**Information acquisition tool**	Hard	Context awareness is obtained solely by hardware
	Soft	Knowledge inference based on context repository is applied to achieve context awareness
**Context model**	Location aware	Context awareness is limited to location awareness
	Medium-level	Various context is used but not unambiguously managed
	Semantic	Semantic technologies are employed to enhance context awareness

The four aforementioned categorizations for context awareness could widen users’ vision to observe context aware systems from different aspects. However, those categorizations differ in their generality and rationality. e.g., it is difficult to attribute a context aware system to a specific level from the aspect of acquisition, because context is obtained from both hard and soft sources in most cases. Similarly, it is blurry to identify the involvement of user interaction as personalized, passive or active. In general, the last classification is the most applicable proposal compared with the others. In Section 3, this classification will be adopted as an important criterion to evaluate different context aware middleware proposals.

### 2.3. Context Lifecycle

**Figure 1:**
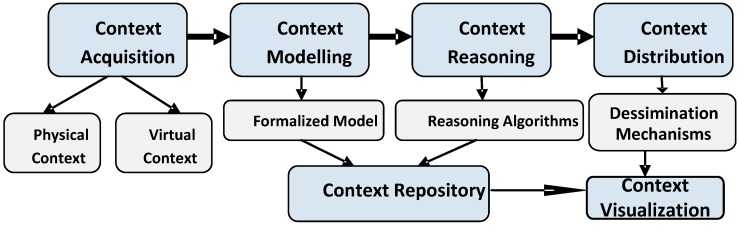
General context lifecycle.

Although current proposals for context aware middleware contain different components or modules to manage context, they obey a general rule which is the context lifecycle. The life of context, which is the period of time from its obtainment to destruction, is demarcated by six significant events as Context Acquisition, Context Modelling, Context Reasoning, Context Distribution, Context Repository, and Context Visualization as illustrated in Figure 1. The way to realize context awareness begins with the acquisition of various kinds of context followed by the formalization and inference process, and finally ends up with the distribution of useful context to the corresponding applications. At the stage of context modelling and reasoning, historical context data needs to be recorded for further use or queries and also can be visualized by users. In the following, the major phases of the lifecycle are outlined in detail.

#### 2.3.1. Context Acquisition

The aim of context acquisition is to obtain a maximum amount of data, such that the possibilities for applications to be intelligent could be maximized due to richer context information. As stated in Table 1, one possibility to classify context is the differentiation into physical and virtual, and the following introduction for context acquisition will be based on this classification.

##### Physical Context

Physical context is obtained from sensing devices which are selected according to the requirements of a certain application or system. It is worth noting that simple sensors (temperature, humidity, *etc.*) and sensor networks are the most widely employed appliance for obtaining context from the surrounding, but with the collaboration of other more complex devices (e.g., Snap2Play [36] and CACH [37]), more types of context can be obtained.

Generally, a sensor is sensitive to only one special phenomenon and monitors some relevant change. Then, it converts the change into data (normally electronic signals). In this way, context consumers are provided with the real time information of a particular property which cannot be obtained directly by observing or touching. However, for detecting all necessary aspects of a certain environment, more sensors are needed. A quite complete list of sensors can be found on the Wikipedia webpage [38].

##### Virtual Context

Virtual context refers to context which could not be sensed (e.g., knowledge, preferences, historical data, estimations, *etc.*) and has to be obtained in different ways. It could be either provided manually or derived from other context. Users serve as an important source and provide a lot of useful personal information, such as birthday, age, preference, height, weight, *etc.* Besides, already obtained context data can be used to infer other context meanings by employing certain reasoning rules.

#### 2.3.2. Context Modelling

In the acquisition phase, a huge amount of context data which is structured in multiple formats is obtained. To make use of them, the premise is to define and store it in a machine readable and processable form, hereby all data should be converted into a unified format such that the context can be understood and shared. This can be achieved by a model that defines, represents and processes the object “context”. 

Many surveys on popular context modelling techniques have been published like e.g., [17,33]. The description for each context modelling technique is quite detailed, however, those surveys are not complete, because some newly proposed modelling techniques are not included, as e.g., multidisciplinary, chemistry inspired *etc.* In the following, ten modelling techniques including “Key-value”, “Markup”, “Graphical”, “Object-oriented”, “Logic-based”, “Multidisciplinary”, “Domain-focused”, “User-centric”, “Ontology-based” and “Chemistry-inspired” will be summarized to give an overview of the most common techniques. The fundamental scheme to examine the available context modelling techniques is based on the data structure used for representation.

##### Key-Value Context Modelling

Key-value pairs are used to enumerate attributes and values in this model. The model can be written in different formats (e.g., text and binary). Because of its simplicity and ease of use, it was widely employed in early research and various service frameworks. For example, Schilit [39] describes the limited number of location information as key-value pairs. However, it lacks capabilities for complex structuring for enabling efficient context retrieval algorithms.

##### Markup Context Modelling

This is referred to as tagged encoding, as context information is stored within tags, *i.e.*, symbols and annotations which represent and format the data. Those symbols and annotations originate from typical markup languages such as XML. Typical representatives of this model are *profiles*. The limit of this model is that its hierarchical structure should be pre-defined and also it is useless to capture context relationships.

##### Graphical Context Modelling

Graphical diagrams enabled by this model are able to specify relationships. Three widely used model examples are Unified Modelling Language (UML), Entity Relationship Model (ERM) and Object Role Model (ORM). The UML is a standardized general-purpose language which expresses different kinds of context information in an own graphical notation, whereas ERM and ORM work for designing and querying databases at the conceptual level. However, the interoperability among different storage databases which are used in the actual low level of graphical model poses a challenge. 

##### Object-Oriented Modelling

The object-oriented model [40] employs class hierarchies and relationships to represent context data and incorporates encapsulation, inheritance and reusability into context expression. Instances can be allowed to access the context by inheritance mechanisms. The core component is called entity and it forms the subject of structured context information. An entity is linked to other entities by means of attributes which are also called associations. This technique stresses developers in terms of being aware of the whole context taxonomy.

##### Logic-Based Context Modelling

In a logic-based model, context is defined as facts, expressions and rules. It is flexible to add, update or remove data in this model. This model thus offers a high degree of formality. A variety of applications have adopted this model. e.g., in [41] a model in a seven field data structure (subject, predicate, object, time, area, certainty, freshness) is developed which helps to organize the information in a sequence. This model is an enabling method to check context consistency and to support the reasoning task as well. However, standards and validation tools are still lacking.

##### Multidisciplinary Context Modelling

This model [42] involves, as the name says, multiple disciplines like psychology, computer science and linguistics. It demonstrates context from different points of view and specifies the relationships among multiple disciplines. The idea is to widen the vision to examine context and to construct a general model, but the complex modelling process introduces difficulties as it incorporates the information concerning many applications, various types of users, and multiple environments. This proposal still remains at the conceptual level. The specific procedures are not clearly figured out and thus the practical usage of this technique is rare.

##### Domain-Focused Context Modelling

Domain-focused context modelling, also referred to as W4 context model, is tailored to model an application domain. Therefore, [43] elaborates the specific mechanism: a four fields tuple [Who, What, Where, When] (the elements are also called knowledge atoms) is recognized in “Someone or something (who) does/did some activity (What) in a certain place (Where) at a specific time (When)”. This model is very expressive and flexible for data usages, and queries, modification and deletion are allowed on context tuples.

##### User-Centric Context Modelling

As the name says, this model is built from a user’s perspective and explores how context information is perceived by users instead of devices, services or applications [44]. Here, “How” and “Why” is added to the formerly presented W4-tuple and leads to the 5W1H-tuple: [Who, When, Where, What, How, Why]. More details about this model are presented in [44]. This method can express context in a very organized way, however, it is just a trade-off between the complexity of expression and ease of use.

##### Ontology-Based Context Modelling

Studer [45] defined ontology as “…*a formal, explicit specification of a shared conceptualization*” in the semantic field. Concepts, instances, and relationships (as main components of ontology) can formally and comprehensively represent the knowledge. This modelling method is regarded to as the most promising method in [17,33], and it can address the conceptual confusion among people and systems because it shares the common understanding. Ontology is competitive over other models in terms of interoperability, formality and reusability.

##### Chemistry Inspired Context Modelling

Ikram *et al.* [46] explored similarity between chemistry and context modelling to fully use chemical reactions and periodic table representation. The general scheme is that context is represented in a reactive model called “Smart Space” [47,48] where associated services can be triggered like chemical bonding or chemical reactions. It is capable of representing various kinds of context and invoking right reactions automatically. However, it is still known by just few people due to its infancy. Besides, it is difficult for the Smart Space model and corresponding graphical visualization to dynamically evolve when the amount and type of context grow.

It is valuable to provide an overview of the introduced models. Therefore, Table 3 summarizes the most important features of every technique such as advantages and disadvantages.

**Table 3:** Comparison of existing context modelling techniques.

Context Modelling Technique	Advantages	Disadvantages	Applicability
**Key-value**	Simple; Ease of use; Flexible	Lack of standards; Useless when big in size; Cannot represent relationships; Difficult to retrieve information; Lack of validation tools; Lack of scalability; Only exact matching.	Adequate to model limited context in simple and self-independent applications which do not need to share data with other applications. Examples: [39,49]
**Markup**	Structured; Some validation tools are available; Flexible.	Lack of standards; Problems in capturing relationships; Timeless; Dependencies; Inconsistency checking; Reasoning and Uncertainty.	Efficient as mode of data transfer about shallow context over network; Applications in which levels of information are few. Examples: [50,51]
**Graphical**	Rich expressiveness; Relationships are allowed; Validation is possible through constraints; Different standards and implementations are available.	Interoperability is unsolved; Configuration must be required; A generic and well-developed standard is needed.	Particularly applicable to derive an ER-model which is useful as structuring instrument for a relational database. Examples: [52,53]
**Object-oriented**	Relationships are allowed; Some development tools are available; Can be fused by using programming languages.	Lack of standards; Lack of validation; Hard to retrieve information; Reasoning is not supported.	Suitable to be used in code-based (high-level programming languages) applications with high computational capability. Examples: [40,54]
**Logic-based**	Rich expressiveness; Support reasoning; Consistency check; Simplicity; Processing tools are available.	Lack of standards; Lack of validation.	Suitable for applications in which high-level information is needed and developers are willing to specify constraints. Examples: [41,55,56]
**Multidisciplinary**	Comprehensive understanding for context based on multiple disciplines; The division of context is concrete.	Too complex; Still at the first stage; Interoperability is unsolved.	Tailored to applications in which key human and social issues should be identified. Examples: [42]
**Domain-focused**	Expressive; Flexible; Structured.	Lack of standards; Lack of validation.	Suitable to single domain-focused applications. Examples: [43]
**User-centric**	Express context in an organized way; Scalability; Allow reasoning.	Lack of standards; Complex to use; Lack of validation; Lack of formality.	Suits applications focused on perspectives of users; data expression is in an intuitive manner. Examples: [44]
**Ontology-based**	Support reasoning; Rich expressiveness; Relationships are allowed; Strong validation; Processing tools available; Mature standards; Interoperability.	Representation can be complicated; It will be complex to retrieve context information; Unable to address uncertainty.	Suitable to applications which highly need to exchange information with others; Sufficient knowledge engineering skills are available. Examples: [45,57,58,59]
**Chemistry inspired**	Medium expressivity to represent many kinds of context; Support for triggering services autonomously; Cross-domain inspired.	Lack of standards; Lack of validation; Not dynamic and scalable; In a nascent stage.	It is possible to apply this model to applications which require spontaneous interaction and composition of information. Examples: [46]

Recalling the introduction for each modelling technique and observing Table 3, it seems obvious that none of the methods is ideal as a standalone technique because all have some limitations. Some of them are only applicable in very simple applications such as key-value and markup methods. The usage of multidisciplinary and chemistry inspired models is considerably limited as they are emerging techniques lacking theoretical support. For the rest of the methods, including graphical, object-oriented, logic-based, domain-focused and user-centric, drawbacks such as lack of interoperability and complexity prevent them from being widely used. The comparison leads to the conclusion that the ontology-based context modelling method could be the most promising technique to model context, because many technical obstacles like interoperability, support for reasoning, strong validation and expressivity are overcome by adopting ontology. However, a fact, that classical ontology is not appropriate to deal with uncertainty, imprecision and vagueness in knowledge, which is inherent to most of real applications [60], should not be negligible. Reference [33] defends that the best way to model context is to create a novel technique to integrate the existing context modelling techniques. For example [61] introduces a hybrid model that combines graphical and ontological techniques, while [62] proposes the integration of fuzzy logic with ontology so as to advance the classical ontology to fuzzy ontology. Since a single model cannot satisfy the requirements arising from complex environments, future efforts could be made to combine different modelling techniques for different purposes in order to mitigate individual shortcomings and fully represent context.

#### 2.3.3. Context Reasoning

Reasoning is also called inference. The demand for context reasoning derives from the fact that context data is imperfect and uncertain by nature [63]. The task of context reasoning is to deduce high level context from raw context associated with some basic functionalities such as validating the context values, filling in missing values, removing outliers, checking context inconsistencies and applying some calculations to obtain new values. In the following subsections, a brief introduction to five popular reasoning techniques is given.

##### Bayesian Network

The core of this technique [64], which belongs to supervised learning, is based on probabilistic reasoning concepts. Entities and relationships among them are represented by directed acyclic graphs and probabilities. Two drawbacks limit its usage: huge demands for exhaustive and exclusive hypotheses and exponential computational overhead.

##### Probabilistic Logic-Based Reasoning

In this reasoning method [65], probabilities are assigned to the context data to make logic assertions. This allows sensor data fusion from two different resources. When conflicts occur, the probabilities can be helpful to make decisions. This technique can only be applied in a scenario with the premise of probability already known. 

##### Case-Based Reasoning 

Context knowledge is deduced from previous similar cases in the case-based reasoning approach [66]. However, it is difficult to accurately calculate the similarity of different cases.

##### Rule-Based Reasoning 

In the rule-based reasoning approach [67], high level information is inferred on the basis of predefined rules. 

##### Ontology-Based Reasoning 

Semantics are incorporated into the reasoning procedure [68]. Based on descriptive logic, it is supported mainly by two semantic web languages: RDF and OWL. However, it is unable to find missing values or accommodate ambiguous information but compensates with expressiveness.

To conclude, the selection of context reasoning technique is subject to two factors: the performance and the requirements arising from the modelling technique used. It is shown that ontology-based reasoning is efficient to deduce high level context because of its predominance of knowledge sharing, logic inference and knowledge reuse. To design a specific application, the selection of the appropriate modelling and reasoning technique should be made carefully taking as many criteria and requirements as possible into account.

#### 2.3.4. Context Distribution

Context distribution is responsible for disseminating useful context information to corresponding applications. Two typical distribution mechanisms (*subscribe/publish* and *polling*) are widely used in current solutions [69].

##### Subscribe/Publish

It is also called *Notification*. Applications interested in certain context information can subscribe to the middleware and be notified when updates of the registered context information occur.

##### Polling

Context consumers are able to actively make queries for their interested context information at any moment. Depending on the used modelling and reasoning techniques, different query methods can be employed. 

#### 2.3.5. Context Visualization

Context visualization offers new ways of seeing data. There is a growing need for an effective visualization to provide a visual overview, explore, analyze, and present phenomena which are often difficult to understand or imagine. Since ontology-based modelling is selected as the best modelling method in the context modelling phase, research on context visualization will be focused on reviewing current approaches to visualize ontology-based context data.

Visualization of ontology-based context data is not an easy task because it means to enrich data with hierarchy, relationships, *etc.* Hence, context visualization ways can be ontology-tailored visualization methods or adapted from other generally used techniques like graph or file system visualization.

In general, ontology-based context data visualization can be grouped into six types: Indented list, Node-link and tree, Zoomable, Space-filtering, Focus + context, and 3D information [70]. Apart from those aforementioned ways, the use of web services and graphical interfaces to present context data is becoming more popular. Different ways of visualization can be adopted according to the users’ preferences. The trend of designing a satisfactory visualization for context should be in line with a more human, interactive, and instinctive manner. Besides, it is worth noting that several properties should be guaranteed in a data visualization approach such as real-time, accuracy and contextual awareness.

## 3. Survey on Context Aware Middleware Architectures

In order to be “smart”, middleware architectures have first to be “aware”. Consequently, apart from a set of general-purposed capabilities, the ability of context awareness is introduced so as to upgrade traditional middleware architectures to context aware middleware architectures. In this light, it is necessary for context aware middleware architectures to provide relevant functionalities to process context concerning the lifecycle of context data. Further, systems or applications built on the top of context aware middleware are able to provide services which automatically adapt to the changing environment.

Indeed, the concept of context aware middleware/systems is not a buzzword emerging in very recent years. It can be traced back to the early 90s, when the first endeavours to develop context aware systems focused merely on exploiting location data, e.g., The Active Badge System [25] and Cricket Compass [71]. Later on, context aware middleware architectures have evolved to achieve more generality and provide support for more types of context information. Many off-the-shelf middleware platforms, including Context Toolkit [72], Gaia [73], Cobra [74], SOCAM [75] just to name a few, can come in handy for developers to build personalized applications. However, they are less likely to be widely used in real implementations due to different limitations, e.g., the adoption of a key-value method in Context Toolkit considerably restricts this middleware to use in very simple applications. Reference [35] claims that early context aware middleware proposals tend to lack several key capabilities such as fault tolerance, semantic interoperability, distributed data computation, and precise reasoning. Along with many technical breakthroughs, such as Cloud Computing, Big Data Analytics, and Artificial Intelligence, in the Information and Communications Technology (ICT) area, the evolution of context aware middleware is also notable. Those aforementioned technologies have favoured the current evolution of context aware middleware. It is thus significant to research the current status of the development of context aware middleware by means of examining the latest middleware proposals.

A holistic view on the latest literature shows that, to organize and evaluate existing context aware middleware, many surveys have been made, e.g., [32,76,77,78]. However, they are largely outdated since all the examined context aware middlewares were proposed before 2010. Two representative ones are shortly outlined in the following.

Kristian [76] presented a survey of a few context aware middleware systems, such as Aura [79], CARMEN [80], CRISMA [81], Cooltown [82], CORTEN [83], Gaia [73], MiddleWhere [84], MobiPADS [85] and SOCAM [75]. A taxonomy of context aware middleware was developed on the basis of several factors such as environment, storage, reflection, quality, composition, migration and adaption. However, these studied middleware proposals are not new and cannot represent the current development status.

Saeed *et al.* [77] gave an overview which contains several typical and old-fashioned middleware architectures. Context aware middleware architectures are analyzed based on several parameters including fault tolerance, adaptability, location transparency, *etc.* However, the focus is placed merely on the comparison of different context aware middlewares. The introduction for each middleware is not substantiated to help readers to get a correct understanding so this survey is not suitable for every kind of reader, and especially it cannot be helpful to beginners without pre-existing middleware experience and knowhow.

As an extension to the aforementioned surveys, the subsequent part of this review aims at providing a critical summary of context middleware architectures proposed during the period from 2009 through 2015 and pointing out potential challenges. Although there is a plethora of so-called context aware middleware proposals in recent the literature, the majority of them are as an afterthought rather than a defined component [86]. More precisely, the overall impression in many proposals is that the context aware middleware layer is not included in the initial design of different context aware systems. Keeping this in mind, eleven well-chosen proposals in terms of defining dedicated context aware middleware architectures are analyzed on the basis of a set of major technical features in the following subsections.

### 3.1. Technical Considerations for Context Aware Middleware

It is feasible to analyze the performance of context aware middleware architectures from various points of view. The following list identifies nine of the most crucial technical attributes for evaluating context aware middleware architectures. In addition, these nine features can also act as the primary considerations at the initial design of context aware middleware architectures.

#### *Architectural Style* 

This defines the way the context aware middleware architecture is constructed. It is the most elementary factor to be initially considered. Four classical architectural fashions can come in handy to organize and arrange the inner composition of middleware.

- The stand-alone architecture is the simplest and least powerful in which only a fixed amount of context is processed in an independent module. Besides, context sharing is not allowed.
- In the layered manner, different responsibilities are allocated to multiple layers. However, the interaction and dependence among all layers raises concerns.
- As the core of the centralized middleware architecture, a central server is endowed with crucial computational capabilities and rich storage capacity. Communication exists between the central server and other devices for exchanging information. The prominent limitation is once the central server fails, the entire architecture will be influenced dramatically.
- The distributed architecture is widely applied and it enables developers to design middleware in a more flexible manner. Different components hold distributed responsibilities and they are independent of each other. However, every component involved in the distributed architecture needs to cope with the stress of computation and storage.

Keeping in mind that the architectural style can considerably affect other significant features such as flexibility and adaptability [77], it is vital to select an appropriate architectural style to conceive the middleware.

#### *Fault Tolerance* 

Fault tolerance means the adaptive capability to respond to unexpected failures. Different middleware may have different reactions to failures. Middleware with high-level fault tolerance can keep the intended operations running and get rid of the influences that failures bring or just be affected in an acceptable degree. Inversely, for some middleware, a minor failure may lead to a sudden stop.

#### *Scalability* 

Scalability is a desired attribute valued in any middleware architecture. Scalability indicates the capability of middleware architectures to accommodate an increasing number of entities, to process a growing volume of work gracefully, and/or to handle a larger scale. Four different types of scalability including load scalability, space scalability, space-time scalability, and structural scalability are summarized in [87]. Since the research of context awareness is the main interest of our survey, the ability to handle an increasing amount of context data will be the exclusive factor to examine if the middleware is scalable.

#### *Security & Privacy* 

Extraordinary focus should be placed on ensuring security and privacy since a lot of sensitive and private data is used. Hence, the middleware should contain security functionalities that can monitor and detect anomalies or unauthorized access to data. Two basic mechanisms can be useful to perform security and privacy functions. On the one hand, context data could be encrypted or authenticated in both the transmission and storage process. On the other hand, access control needs to be clearly identified.

#### *Interoperability* 

The definition of interoperability is “*the ability of two or more systems or components to exchange information and to use the information that has been exchanged*” [88]. Two possible types of interoperability can be achieved. On the one hand, inner components can interact with each other and share information. On the other hand, different context aware middleware can communicate with each other and make use of exchanged information.

#### *Service Discovery* 

Lots of benefits can be obtained by means of service discovery, such as discovering, combining and orchestrating services. Repositories can be exploited to store corresponding service profiles including attributes, parameters and locations for further queries, the location of repositories can either be centralized or distributed. Some service discovery protocols are available, e.g., the Simple Service Discovery Protocol (SSDP) [89] or Service Discovery Service (SDS) [90].

#### *Context Abstraction* 

Context abstraction explains how context is expressed and formalized. The increase of the abstraction level can improve the ability of reading, understanding and using context. Besides, it can be beneficial to the reasoning procedure.

#### *Storage* 

The storage of context is highly demanded as historical context is still meaningful for further use. Based on the context trend, predictions for next actions can be made. The history of context is a good knowledge source for the reasoning process. Taking into account the huge data volume, an appropriate storage container should be carefully chosen.

#### *Cloud-Based Big Data Analytics* 

According to IBM data scientists, four characteristics (the 4 V’s) can be used to define the concept of big data: volume, variety, velocity, and veracity [91]. Due to the nature of context data, context can fit into the definition of big data and be regarded as big data. Context data is of massive volume and presents a big variety of types. Besides, it changes continuously in terms of velocity and its veracity can also be satisfied since it can accurately represent the real changes of involving circumstance. Therefore, “big” context data introduces new challenges but also brings new capabilities to context aware middleware architectures. The realization of context awareness can be supercharged by existing big data techniques such as massive parallel and in-memory databases, deep packet inspection technology *etc.* Here, the outstanding technology that could considerably drive context aware middleware from holding “big data” to “big wisdom” is cloud-based big data analytics. It can enable context aware middleware to access needed computing resources from the cloud computing, foresee trends over big data and mine more useful information for context aware decision making.

### 3.2. Context Aware Middleware Architectures

The following list contains 11 new and representative context aware middleware architectures proposed during the period from 2009 through 2015 to find the newest development tendency.

#### 3.2.1. Context Aware Middleware for Pervasive Elderly Homecare (CAMPH)

CAMPH [92] is a middleware to glue hardware infrastructure with various context aware applications, especially with emphasis on the pervasive homecare area. Generally, this middleware offers several key-enabling system-level services, including context data acquisition, context storage, context reasoning, context query processing, service organization and discovery. The overall architecture consists of four logical layers: *Physical Space Layer*, *Context Data Management Layer*, *Service Management Layer*, and *Application Layer*.

- *Physical Space Layer*: Each Physical Space (PS) may contain physical entities such as sensors, actuators and computing devices. At this layer, data is modelled as a pair of attribute-value. PSs with high relevance, such as having similar attributes, are sorted into the same class named *context domain* or *context space*.
- *Context Data Management Layer*: Main components allocated in this layer are Context data/events database, Query processing, Context space management, and Context reasoning. A hierarchical reasoning scheme, in which low-level reasoning performs on single PS data while high-level reasoning makes inference from context domain, is applied. Besides, SQL-based context query interface is available to acquire context data or subscribe to event notifications.
- *Service Management Layer*: Context data is fully utilized to enable context aware service organization and discovery.
- *Application Layer*: Different homecare applications can invoke and orchestrate context aware services or make requests for context data directly in this layer.

An example of a personalized homecare application mashed from web services running this middleware was developed to demonstrate the working principles of CAMPH. However, this prototype is still far from usable. The usability of CAMPH should be examined in a larger scale field trial. Besides, context data exchangeability/interoperability among different context domain is a concern since context data is structured in key-value model. How to manage the massive amount of context data from various spaces is still an unsolved issue. Although it is declared that a hierarchical and comprehensive reasoning scheme will be deployed, explanation about detailed procedures is still missing. Security and privacy are not considered during the entire conception/design.

#### 3.2.2. ACoMS+

ACoMS+ [93], as an enhancement of ACoMS [2], offers a solution of resource efficient and context aware management of sensing infrastructure. The core of ACoMS+ is composed of a *Context Source Manager* (models raw context information and performs actions on low-level communication), an *Application Context* *Subscription Manager* (allows applications to subscribe interested context by specifying quality of information and service), and a *Reconfiguration Manager* (reconfigures sensing devices to offer fault-tolerant provisioning of information). Context Modelling Language (CML) is selected as the modelling method which leverages the graphical notations to represent context information.

To make the reconfiguration process more elaborate, a mining algorithm called HiCoRE [94] is incorporated into ACoMS+. While dealing with different applications, decisions can be made wisely based on those correlated rules of context which are mined by HiCoRE. ACoMS+ adopts the HiCoRE algorithm to fully utilize operational objectives and discover correlations among sensing infrastructure. However, HiCoRE is able to mine and rank correlations on the premise that every sensing entity can only perform a single sensing aspect (such as temperature, humidity). Future efforts can be focused to make the mining algorithm more comprehensive by taking into account complex sensing devices (e.g., camera, tablet) with multiple sensing capabilities at the same time. Besides, the presented design has just been partially implemented and tested. Continuous research is still needed for improving this proposal.

#### 3.2.3. Octopus

*Octopus* [95] is conceived as an open-source, dynamically extensible middleware to facilitate smart home/office domain-specific applications. *Octopus* is constructed in a simple layered architecture while it is ambitious to tackle two technical problems: data management and data fusion. Five distinguishable layers form the whole infrastructure: *Sensing layer*, *Aggregation layer*, *Analysis layer*, *World model layer*, and *Application layer*. The *World model layer* builds a *world model* to insert all context data abstracted from *Aggregation layer* to predefined objects. Multiple tiers of *solvers* run in the *Analysis layer* to process context information, such as generating high-level information from lower-level world model information. *Solver*, as an independent module, can be added into or removed from the middleware based on different requirements. Multiple *solvers* can be integrated to execute more complex operations.

It is claimed in [95] that *Octopus*, which has been deployed on two different university campuses and tested for over 6 months, is simple-yet-powerful. However, it lacks proof of concept since the implementation details and also evaluation results have not been demonstrated to the public.

#### 3.2.4. FIWARE

The FIWARE [96] FP7 project has an ambitious intention to strengthen the competitiveness of the EU economy by presenting a cutting-edge infrastructure in which creation and delivery of services, high QoS and security are enabled. This platform is conceived to be considerably generic and could adaptively fit into various usage areas, e.g., safety, logistics, environment, energy, traffic and mobility, and agriculture. This platform is built based on public cloud with a rich library of modules offering various added-value functions (referred as services). These modules, regarded as Generic Enablers (GEs), fulfil all the capabilities of different chapters of this architecture, such as service delivery, cloud hosting, Internet of Things, support services, developer tools, and interface to the network and devices. Among all, it is worth stressing that enablers dedicated to manage context data from heterogeneous resources are playing an important role in the whole platform. These aforementioned enablers could be grouped into two categories: the Semantic Virtualization Enablers and the Cognitive Enablers.

Three main concepts, which are *Actors*, *Resources*, and *Applications*, are virtualized so as to lay the basic foundation of semantics of this cognitive middleware. The Semantic Virtualization Enablers are responsible for abstracting the heterogeneous Actors, Resources, and Applications by means of attaching homogeneous, context aware and semantic aggregated metadata. On the basis of semantically abstracted metadata available through well-defined Restful APIs, Cognitive Enablers are capable of making decisions regarding the best solution of exploiting the available resources to efficiently satisfy *Application* requirements and needs. To meet a specific application’s needs, different Cognitive Enablers can be dynamically orchestrated. In the real deployment, all the functionalities of enablers can be distributed into different physical network entities.

The design of the Semantic Virtualization Enablers and Cognitive Enablers allows *Applications* to transparently, efficiently and flexibly employ available Resources and become customer-tailored. With the collaboration of other Enablers such as Security Enablers, Cloud Hosting Enablers and IoT Enablers *etc.*, the proposed FIWARE is upgraded to be a full-fledged middleware with context awareness, interoperability, cloud hosting, big data analytics, and service delivery and composition. What’s more appealing for developers is that, so far, all the Enablers developed in the FIWARE project are available as open-source implementations associated with detailed user manuals. However, it still will not be an easy task to move the pilot tests to real commercial usages. More potential constraints introduced by harsh environments such as underwater and off-shore should be taken into account when adapting *FIWARE* to actual operations.

#### 3.2.5. Context Awareness for Internet of Things (CA4IOT)

**Figure 2:**
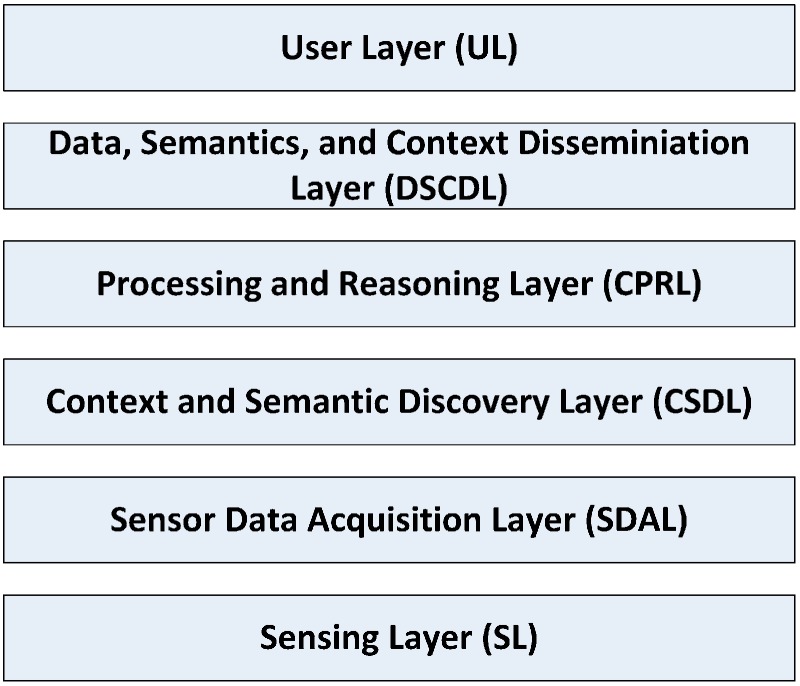
CA4IOT architecture, as described in [97].

A sensing-as-a-service middleware presented in [97] is called CA4IOT. It appears that this middleware is conceived merely to solve a single issue of how to select the most suitable sensors according to the tasks/problems at hand rather than providing a complete middleware solution for managing context data. An overview of CA4IOT architecture is displayed in Figure 2.

Four major layers form the middlew are which are listed as follows:

- *SDAL*. The main components located in this layer are sensor wrappers, wrapper repository, wrapper generator, sensor device definition (SDD) local repository and SDD cloud repository. This layer is responsible for acquiring a variety of context data.
- *CSDL*. This layer is in charge of discovering context and semantic. Relevant components are context and semantic discoverers, context and semantic discoverer generator, and context and semantic discoverers repository.
- *CPRL*. A collection of important functions are distributed in this layer like processing data, reasoning high-level context, fusing context, knowledge generating and storing.
- *DSCDL*. Users can make requests via multi-model interfaces. Local repository can interact with repositories which reside in the cloud or open linked data to provide better answers for those queries by means of big data analytics.

In the *CSDL* and *CPRL* layers, context data related to sensors are represented in XML. In addition, users submit their requests for querying context using XML data format. In general, this middleware is quite complex and mature with detailed explanations for different functional component. Many technical factors such as context abstraction, context process and context dissemination are carefully considered while a significant technical consideration which is security and privacy are missing in this middleware. Compared with other middleware solutions, this middleware can act as either a standalone middleware or an auxiliary technique to be integrated with other framework solutions to fulfil the demands from different paradigms. However, only a simple use case is employed to show the specific procedures of developing the proposed middleware, and implementation of this middleware is still missing.

#### 3.2.6. CAMPUS

CAMPUS [98], short for Context-Aware Middleware for Pervasive and Ubiquitous Service, is proposed to automate context aware adaptation decisions with the influence of three key technologies: compositional adaptation, ontology, and description logic/first-order logic reasoning. It has taken an enormous step to advocate automated run-time adaptation decisions instead of depending on predefined adaptation policies that only take limited contextual changes potentially operating in a dynamic situation.

CAMPUS, as a typical layered architecture, consists of three tiers: *the programming layer*, *the knowledge layer*, and *the decision layer*.

- *The programming layer*. It is responsible for constructing and reconfiguring context aware applications by adopting the instructions from the decision layer.
- *The knowledge layer*. Three ontologies including Context Model, Tasklet Model, and Service Model are proposed to represent semantics of knowledge which is necessarily required by *CAMPUS* to make adaptation decisions. The knowledge could be the requirements desired by target service, the properties of the available tasklets, the context requirements imposed by tasklets, and the properties of run-time context.
- *The decision layer*. Decision maker uses a multi-stage normative decision model, which includes preprocessing, screening and choice, to choose the best tasklet alternatives for a given task. The automated adaptation decisions will be forwarded to the programming layer.

CAMPUS provides an effective middleware solution for integrating context awareness to application development. CAMPUS could automatically derive context-aware adaptation decisions at run time by means of semantic-enhanced decision making. The initial implementation of CAMPUS is built in Java SE 1.6 along with additional plug-ins such as Pellet 1.5.1 for description reasoning and Jess 7.1p2 for logical reasoning. However, security has not been mentioned in this proposal and collaborative decision making among multiple CAMPUS middleware instances can be a future extension.

#### 3.2.7. Context-Aware Services Framework (CASF)

**Figure 3:**
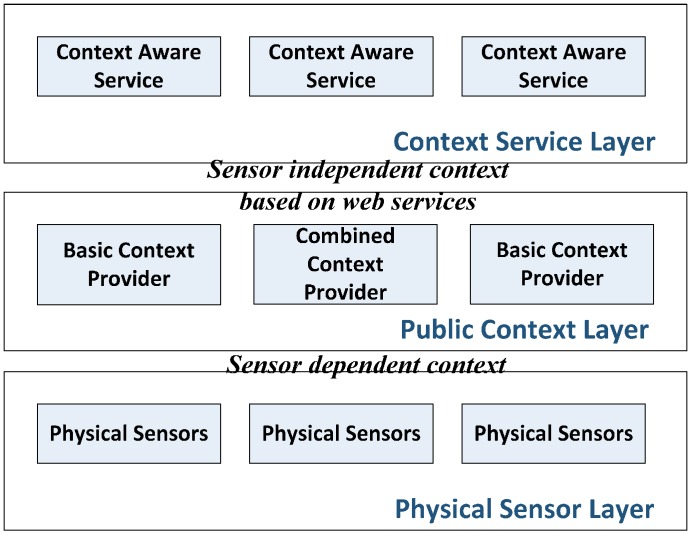
CASF, as described in [99].

A middleware proposal, here abbreviated as CASF, aiming at providing a variety of context aware services was presented in [99]. The authors Juyoung *et al.* realized that many context aware middleware architectures lack service discovery and composition capability. Consequently, they came up with this new architecture aiming at tackling this gap. Basically, this framework is built based on semantic web services as they are well-known for supporting automatic service discovery and integration. To achieve the integration of services, which also refers to as selection and combination of context information, this proposal separates context aware services with context aware information. The core of this architecture, named as context mediation framework, is shown in Figure 3.

The context mediation framework consists of three different tiers: *physical sensor layer*, *public context layer* and *context service layer*.

- *Physical sensor layer*. It can only recognize sensor data. Physical sensors are the only context information source.
- *Public context layer*. Two types of context providers in terms of complexity of context information process are located in this layer. A basic context provider only processes sensor data from physical sensors while a combined context provider can make use of information from both sensors and other context providers. All context information generated in this layer is served based on web services so that openness and interoperability are achieved. By using a proposed context ontology and OWL-S, context providers are able to be constructed in web services.
- *Context service layer*. Context information is consumed in this layer so that context aware services can be generated and provided to users.

The major novelty of this proposal is the adoption of the concept of semantic web services. By publishing context information based on semantic web services, it is also feasible to achieve automatic discovery and integration for context information. However, many follow-up studies should be conducted to make the proposal complete. Firstly, more detailed protocols and ontologies should be specified to translate context information to web services. e.g., SOAP-based messaging protocol could be adopted to connect the communication between public context layer and physical sensor layer with more details explained. Besides, there is still lack of implementation, therefore evaluation. This architecture lacks prototyping test by building various advanced context aware services with regard to real environments.

#### 3.2.8. Semantic Web-Based Context Management (SeCoMan)

**Figure 4:**
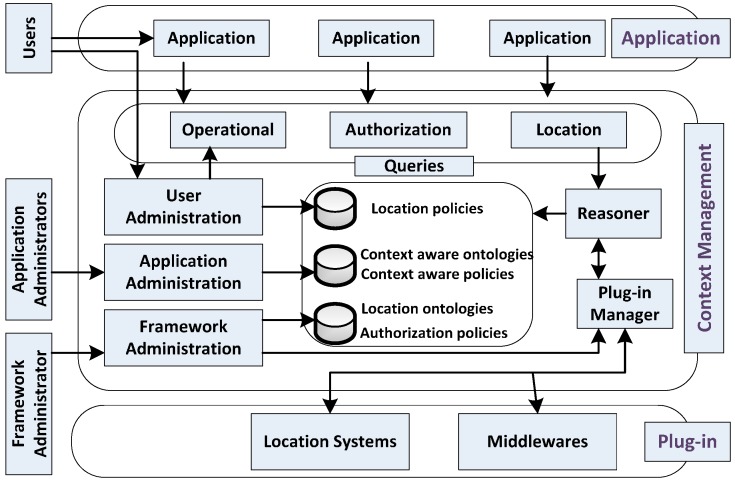
SeCoMan, as described in [100].

SeCoMan [100], as the abbreviation of Semantic Web-based Context Management, is intended to provide a privacy-preserving solution for developing context-aware smart applications. In SeCoMan, ontology is employed to model the description of entities, reason over data to obtain useful knowledge, and define context-aware policies. The whole architecture of SeCoMan is shown in Figure 4.

All the modules making up SeCoMan are placed in a layered structure including *Application*, *Context Management*, and *Plug-in* (from top to bottom).

- *Application*. Different applications reside on top of SeCoMan in order to offer desired services for users.
- *Context Management*. As the core of the SeCoMan framework, it provides context aware supports for applications. Three kinds of actors with different rights to interact with SeCoMan are defined including *Framework Administrator*, *Application Administrator*, and *Users*. A set of predefined queries are allowed for applications to get information about indoor location of users and objects. Semantic rules are used to specify policies regarding restricted access to location information so that privacy is guaranteed.
- *Plug-in*. It provides SeCoMan with context information, which is especially focused on locations. In other words, the plug-in layer acts as an independent context source.

In fact, the so-called context aware solution offered by *SeCoMan* is limited to get aware of locations. Therefore, privacy protection is fulfilled in a location-limited level which enables users to share their location with the right users, at the right granularity, at the right place, and at the right time. *SeCoMan*, especially the context management layer, is planned to be integrated in the cloud architecture in future work. In this way, this middleware could take advantage of the features of cloud computing to achieve extra capabilities, such as elasticity, monitoring, load balancing, and address security issues. Besides, the current privacy schema will be augmented by introducing anonymity and hashing policies to hide and disguise the identity of a user. The exploration of outdoor usage could be the next step to improve the generality of this middleware.

#### 3.2.9. CoCaMAAL

Forkan *et al.* [101] presented a novel Cloud-oriented Context-Aware Middleware in Ambient Assisted Living (AAL) which is abbreviated as CoCaMAAL. The motivation behind is that biomedical sensors which are widely used in AAL lack the processing power to perform key monitoring and data-aggregation tasks. Therefore, cloud computing is adopted to address computing needs. In particular, this proposal is believed to serve as a scalable and context aware framework which can ease the flow between data collection and data processing in AAL scenarios. Basically, CoCaMAAL is built on the basis of Service-Oriented Architecture (SOA) which performs context modelling for raw data, context data management and adaption, context aware service mapping, service distribution, and service discovery. CoCaMAAL comprises five main cloud-oriented components: *AAL systems*, *context aggregator and providers (CAP) cloud*, *service providers cloud*, *context aware middleware (CaM) cloud*, and *context data visualization cloud*.

- *AAL systems*. This component, as the hardware architecture, includes different BSN (Body Sensor Network) foundations and monitoring systems for meeting different target user requirements.
- *Context aggregator and providers (CAP)*. Raw data from AAL systems is converted and abstracted to high-level context by *CAP*. More specifically, context providers categorize sensor data into context based on pre-designed ontology. Afterwards, different context is integrated by context aggregator to provide complete information. In addition, reasoning mechanisms are applied to infer more useful information.
- *Service providers*. They are the producers of context aware services, such as applications.
- *Context aware middleware (CaM)*. By utilizing existing knowledge and incoming context, CoM is able to indentify assistive services for the given context and trigger associated actions. CaM is the core component of CoCaMAAL with multiple key functions, such as context management, context storing, context retrieval, context manipulation, service mapping, self-adaptation, service discovery, and security service.
- *Context data visualization*. Proper interfaces (e.g., GUI) are available for users to visualize context data.

A prototype based on CoCaMAAL was developed in Java. The implementation examines the performance of the proposed architecture, such as the influence of increasing context and service load on service response time. The results prove that CoCaMAAL is efficient at collecting, abstracting, and using context from AAL environments to provide context aware services. The major novelty is the adoption of cloud computing which provides powerful computing capabilities to process context. However, several concerning issues cannot be ignored. e.g., conflicts in context are not considered and reliability analysis is not accomplished. Although [101] states that the context aware role-based access control and privacy-preserving context service protocol can be adopted to ensure privacy in this middleware, those two mentioned approaches are not included in the test.

#### 3.2.10. Big Data for Context Aware Monitoring (BDCaM)

Motivated by CoCaMAAL, a novel context aware middleware architecture, named Big Data for Context-aware Monitoring (BDCaM) [102], is proposed. As an extension of CoCaMAAL, BDCaM addresses additional concern: personalized knowledge discovery. The underlying approach of discovering personalized knowledge is to derive/learn patient-specific anomalies from amounts of data. The adoption of a novel learning process in BDCaM is an important step forward. A 2-step learning methodology is newly proposed in [102] to derive more useful information for context aware decision making. The specific procedure of this learning approach is as follows: firstly, correlations between context attributes and threshold values are identified. Possible association rules which are patient-tailored will be generated by applying the MapReduce Apriori algorithm [103]. Finally, supervised learning is performed over context data based on those rules generated in the first step. Like CoCaMAAL, BDCaM is split into several distributed and cloud-based components which are Ambient Assisted Living (AAL) Systems, Personal Cloud Servers (PCS), Data Collector and Forwarder (DCF), Context Aggregator (CA), Context Providers (CP), Context Management System (CMS), Service Providers (SP), and Remote Monitoring Systems (RMS). A use case related to health monitoring is implemented on this middleware and implementation results have proven the applicability of this middleware and the efficiency of detecting patient's anomalies. However, security and privacy for personal data is still lacking in this middleware. Besides, exploring the possibility of generalizing this middleware to suit more domains (not limited to AAL) could be a further improvement.

#### 3.2.11. FlexRFID

A recently published proposal called FlexRFID [104] aims to provide a policy-based middleware solution for facilitating the development of context aware applications and integrating heterogeneous devices. Ponder is adopted as the policy specification language in this middleware. The FlexRFID middleware is a multi-layered middleware consisting of *Device Abstraction Layer* (*DAL*), which abstracts the interactive operations among the physical network devices), *Business Event and Data Processing Layer (BEDPL)*, it provides context data management like aggregation, transformation and dissemination), *Business Rule Layer* (*BRL*, it manages policy-related operations), and *Application Abstraction Layer* (*AAL*, it enables communications among applications and the FlexRFID). FlexRFID is claimed to provide all data processing capabilities like filtering, grouping, dissemination and duplicate removal. In addition, it is an enabling solution to support simultaneous communication among different applications which are built in this middleware. Notably, FlexRFID differs from other context aware middleware in the capability of policy enforcement. A plethora of benefits can be achieved by defining different types of policies such as ensuring privacy, constraining access control, and offering customized services. Two abstract types of policies are stated in FlexRFID: System Policies (manage the operations done by the middleware) and Application Policies (define the way users want the FlexRFID services to be delivered).

The authors in [104] focus mostly on implementation details and performance evaluations of FlexRFID. Experimental results obtained from two real scenarios (healthcare and book management) show that the response time will become longer as the volume of policies increases. Also for this reason, some specifications for the concrete techniques used in the middleware are missing in this paper. For example, a data formalization method is not included. The current version of the FlexRFID middleware only offers basic security mechanisms by means of specifying access control policies. More advanced security measures should be taken, e.g., application authentication and security at the level of tags and sensor nodes. Further improvement could be integrating FlexRFID in the cloud so as to enable applications to flexibly use cloud-based services and adapt those services with regard to specific application policies and context considerations.

### 3.3. Comparisons

After presenting the chosen set of context aware middleware, it is valuable to analyze and evaluate each of them. Table 4 and Table 5 present the comparative results of the previously investigated architectures with regard to many parameters as publishing year, architectural style, context abstraction, context reasoning, scalability, cloud-based big data analytics, fault tolerance, interoperability, service discovery, storage, security and privacy and context awareness level. The list of context aware middleware is ordered based on the chronological sequence ranging from 2009 through 2015.

**Table 4:** Comparison of context aware middleware architectures (Part I).

Middleware	Year	Architectural Style	Context Abstraction	Context Reasoning	Scalability	Cloud-Based Big Data Analytics
*CAMPH*	2009	Layered & Distributed	Key-value	Rule	×	×
*ACoMS+*	2010	Distributed	Graphical	Rule	√	√
*Octopus*	2011	Layered	Not specified	Not specified	×	×
*FIWARE*	2011	Distributed	Ontology	Ontology	√	√
*CA4IoT*	2012	Layered & Distributed	Ontology	Ontology and statistical	√	√
*CAMPUS*	2013	Layered	Ontology	Ontology	×	×
*CASF*	2013	Layered	Ontology	Ontology	×	×
*SeCoMan*	2014	Layered	Ontology	Ontology and rule	×	×
*CoCaMAAL*	2014	Distributed	Ontology	Ontology	√	√
*BDCaM*	2015	Distributed	Ontology	Ontology	√	√
*FlexRFID*	2015	Layered & Distributed	Markup	Rule	×	×

**Table 5:** Comparison of context aware middleware architectures (Part II).

Middleware	Fault Tolerance	Interoperability	Service Discovery	Storage	Security & Privacy	Context Awareness Level
*CAMPH*	×	×	√	√	×	Medium level
*ACoMS+*	√	×	√	√	×	Medium level
*Octopus*	√	×	×	√	×	Medium level
*FIWARE*	√	√	√	√	√	Semantic
*CA4IoT*	√	√	√	√	×	Semantic
*CAMPUS*	×	√	√	√	×	Semantic
*CASF*	×	√	√	√	×	Semantic
*SeCoMan*	×	√	×	√	√	Location aware
*CoCaMAAL*	√	√	√	√	×	Semantic
*BDCaM*	√	√	√	√	×	Semantic
*FlexRFID*	√	×	√	√	√	Medium level

As revealed from Table 4, layered, distributed or layered snd distributed have been popular architectural fashions for recent middleware proposals. The trend to organize middleware compositions tends to be more flexible and reliable while in the early stage the majority of middleware architectures such as *Cobra* [74] and *MoCA* [105] adopted a centralized approach. Structured in a layered or distributed manner, middleware architectures could outperform centralized middleware in terms of extensibility. It is more feasible for layered or distributed middleware to allow the build-up of additional features or capabilities with minimum effort (e.g., without major redesign, with minimum impact on existing components) in order to fulfil a very specialized purpose. In a layered middleware architecture, each layer uses the previous layer to implement new functionalities that will be exported to the layer above. In this way, distributed middleware architectures outperform layered ones in terms of reliability due to the fact that layered middleware architectures are prone to errors because of lower layers’ failures. The layered & distributed combined manner allows developers to easily manage the software construction with intuitive conceptual understanding and functionality distribution in a hierarchical (layer by layer) and distributed (inside every layer) way.

Undoubtedly, ontology has dominated the landscape of modelling context data in all investigated proposals except CAMPH, ACoMS+, Octopus and FlexRFID. Extra features are enforced by the adoption of ontology such as high-level context abstraction, powerful reasoning, semantic interoperability, and (probably) advanced context awareness. In ontology-based context aware middleware architectures (e.g., FIWARE, CA4IoT, CAMPUS, CASF, SeCoMan, CoCaMAAL and BDCaM), different ontologies are proposed and used to unambiguously formalize the knowledge base so that all entities within/upon the middleware could share the same understanding. However, it could raise a concern when different instances of different middleware want to cooperate and exchange information. For this reason, ontology mapping/alignment among various ontologies employed in different middleware should be discreetly addressed.

Reasoning is a crucial factor for inspecting the middleware performance due to its considerable contributions to improve the awareness and smartness of middleware. Although ontology is the primary choice to infer useful information, it can be predicted that hybrid reasoning models will be preferred over ontology in the foreseeable future. For example, SeCoMan proposed a hybrid model which combines ontology and rule. This hybrid model enables user-defined rules via Semantic Web Rule Language (SWRL) as well as description logics.

As the judging factor for scalability is intensively focused on the capability of handling context data, the scalability attribute of middleware architectures in this survey is considerably subject to the fact whether cloud-oriented techniques are employed. In this light, cloud-oriented techniques are an elegant solution to solve the scalability issue from different aspects. More specifically, in *ACoMS+*, a mining algorithm derived from cloud-based big data analytics is exploited to reconfigure existing resources to operate efficiently in small-scale and large-scale environments. Other middleware proposals including FIWARE, CA4IoT, CoCaMAAL, and BDCaM achieve multiple benefits from cloud. They extend data storage by means of cloud repository, enhance processing capability by means of cloud computing, and mine data by means of big data analytics. In addition to this, *FIWARE* expands its exploration on cloud-oriented techniques to a greater extent, such as visualization of data and invoking cloud-services from different providers.

Currently, in most proposed solutions, the functionalities of middleware are achieved by distributing the tasks in a layered/distributed architecture. There is a trend to leverage cloud resources to design an efficient and solid middleware. However, to “shift” middleware to the cloud will exert more stress on the security and privacy issue. In fact, as seen in Table 5, only three middleware architectures take security and privacy into account by applying different strategies. e.g., *FIWARE* developed a set of key enablers aiming at guaranteeing security privacy and trust from two levels: generic and optional. In SeCoMan, users’ privacy is enforced by adding anonymity and hashing policies to hide and disguise the identity of a user. FlexRFID offers basic support for security through the use of access control policies.

Service discovery is missing in *Octopus* and SeCoMan. Mechanisms to discover services in other middleware are various. e.g., CAMPUS contains a component called Service Directory acting as a reference to all available services. More specifically, *Service Directory* is in charge of registering and locating all services provided by the middleware so that context consumers could find the services needed. In *CASF*, it is possible to search for and combine services which are suitable for the user’s purpose by utilizing Semantic Web Services. Forkan *et al.* [101] declare that CoCaMAAL allows service discovery to build compound services. However, the statement is still blurry and cannot be proven without specifying detailed methodology.

As demonstrated from Table 5, all middleware architectures provide technical suggestions for storing historical context. Having a deep look at storage means, it can be found that cloud-based middleware architectures (ACoMS+, FIWARE, CA4IoT, CoCaMAAL and BDCaM) utilize distributed cloud repositories for storing context while other proposals keep context in a local knowledge base.

As far as context awareness is concerned, different levels are reached by different middleware. Since *SeCoMan* is dedicated to provide services according to location changes, it is limited to location aware. The highest context awareness level is achieved by FIWARE, CA4IoT, CAMPUS, CASF, CoCaMAAL and BDCaM.

## 4. Open Issues

Several technical challenges have been detected in the presented middleware architectures. The most relevant and crucial open issues are presented as follows:

- *Security & Privacy* are a must in any middleware architecture. Amounts of sensitive context are employed to characterize the situation so as to provide relevant services. Hence, the demand for enforcing security & privacy is increasing. Besides, as it is agreed that it will be a fashion to drive middleware architectures to be cloud-oriented, leveraging cloud resources will impose many challenges on security & privacy. e.g., it could be possible for any entity with internet connection to easily collect, access, visualize, archive, share, and search data or services from the cloud. It will not be an easy task to guarantee security & privacy, political or legal restrictions of data in cloud. Potential solutions could be restricting unauthorized manipulation, protecting privacy of information storage, ensuring the information security during processing or probably creating private clouds dedicated to public sectors.
- *Increase the degree/level of context awareness*. There has been a plethora of so-called context aware middleware proposals in recent years. However, they differ in real capabilities, which refer to context awareness level. The desired awareness means that middleware could adequately understand any change of current environment based on all available context information. The majority of current context aware middleware proposals only reach a very limited level of cognition and awareness for their involved circumstances.

More efforts should be put to drive the current middleware proposals towards better ones with higher level of context awareness.

- *Standardization*. It seems that it will not be feasible to formulate a single standard for a generalized context aware middleware due to the variety of domains and applications involved. However, considerable efforts have been put to build a generic domain-focused middleware solution [106]. For example, [107] put forward a standardized middleware for the semantic web domain. Different middleware solutions adopt different standards compliant with needs from different domains. All these standards should be collected to form a complete standardization platform in which the selection of standard could be enabled to fit in a certain domain.
- *Increase autonomy*. Although context aware middleware architectures reduce the need of human intervention when they serve personalized applications, human intervention is still necessary and playing an important role in realizing context awareness. For instance, to infer more useful and higher-level context information, users have to define rules again and again when the involved surrounding changes. Real autonomy can be developed if the inference rules can evolve and change automatically according to the changing environment. Potential solutions can make use of big data related techniques like mining and learning algorithms.
- *Lack of testing*. It can be noted that most of middleware architectures included in this survey are still at the conceptual stage. Prototypes based on some of these middleware solutions have been built, but complete implementations (let alone actual usage) are missing. Besides, commercial or practical operation which goes beyond simple pilot projects is even a more difficult target. More attempts should be made to drive the move of these middleware solutions from theoretical research to tests and further to full-fledged deployment to actual environments.

## 5. Conclusions

This paper has presented a review on the latest prominent solutions for context aware middleware during the period from 2009 through 2015. Two major contributions have made as follows: 

Firstly, a preliminary background about the general approach of realizing context awareness has been presented to provide a starting point for readers to understand context aware middleware. Herein, the principal terminologies of context, context awareness and their roles in context aware middleware architectures have been introduced. Then, four different classifications of context awareness levels have been reviewed. After a thorough analysis, the categorization which divides context awareness into three levels: location aware, medium and semantic has been chosen as the most reasonable and appropriate criterion to evaluate context aware middleware architectures. An extensive and comprehensive study on existing context modelling and reasoning techniques has been carried out with basic explanations and comparison. The aforementioned study is able to give an insight into the strength and weakness of each modelling and reasoning technique which could help users select the most suitable one for their own use.

Secondly, a state-of-the-art overview on eleven representative context aware middleware architectures from 2009 to 2015 has been conducted. Several technical considerations for designing context aware middleware architectures have been discussed. The eleven selected context aware middleware architectures have been introduced and analyzed in depth. Furthermore, a taxonomy of these studied middlewares has been worked out with regard to different evaluation factors. Comparative results have revealed that there is no context aware middleware architecture that is suitable for “all settings”. Hence, the comparison of the existing middleware architectures is significant to make users aware of the advantages and disadvantages of each middleware, thus to choose the most suitable solution for a specific need. Based on the proposed taxonomy, five challenges observing from the studied middleware proposals have been pointed out. Therefore, future work could be focused on solving limitations in the current middleware and probably proposing a new context aware middleware solution.
